# THE POWER OF DAUGHTERS: THE ROLE OF SIBSHIP GENDER COMPOSITION IN MOTHER-ADULT CHILD RELATIONS

**DOI:** 10.1093/geroni/igac059.2338

**Published:** 2022-12-20

**Authors:** Robert Frase, J Jill Suitor, Megan Gilligan, Reilly Kincaid, Catherine Stepniak

**Affiliations:** Purdue University, West Lafayette, Indiana, United States; Purdue University, West Lafayette, Indiana, United States; Iowa State University, Ames, Iowa, United States; Purdue University, West Lafayette, Indiana, United States; Purdue University, West Lafayette, Indiana, United States

## Abstract

Prior scholarship demonstrates that older mothers receive more care from daughters, prefer daughters as caregivers, and have stronger emotional bonds with daughters. Despite these clear gendered differences in care, care preferences, and closeness, less is known about whether the presence of daughters in a family affects mother-adult son relationships or whether the presence of sons in a family affects mother-adult daughter relationships. Drawing from theories of gender socialization and social exchange, we propose that mothers would, given the choice between daughters and sons, prefer to receive care from and engage in emotional exchanges with daughters. Therefore, we predict sons’ care to and emotional closeness with older mothers will be inversely related to the number of daughters in the family. We test our hypotheses with mixed-method data from 1,577 mother-adult child dyads nested within 420 families collected as part of the Within-Family Differences Study-II. Findings support our hypotheses. The larger the number of daughters, the less likely sons are to provide care to their mothers, whereas the likelihood of daughters providing care is unaffected by the number of sons. Similarly, the larger the number of daughters, the lower closeness mothers report with their sons, whereas mother-adult daughter closeness is unrelated to the number of sons. In sum, our findings show ways in which both an adult child’s gender and the gender composition of their sibship affect mother-adult child relationships, as well as highlighting the applicability of theories of gender socialization and social exchange to the context of aging families.

